# Rotavirus Vaccines: a story of success with challenges ahead

**DOI:** 10.12688/f1000research.11912.1

**Published:** 2017-08-18

**Authors:** Miguel O’Ryan

**Affiliations:** 1 Institute of Biomedical Sciences and Millenium Institute of Immunology and Immunotherapy, Faculty of Medicine, University of Chile, Santiago, Chile

**Keywords:** rotavirus, vaccination, gastroenteritis, immunity

## Abstract

Approximately 40 years have passed since the discovery of the rotavirus and 10 years since the introduction and progressive dissemination of rotavirus vaccines worldwide. Currently, 92 countries have introduced rotavirus vaccines into national or subnational programs with evident impact in disease reduction. Two vaccines have been widely used, and four additional vaccines have been licensed and are being used in defined regions. In this context, one main issue that remains unsolved is the lower vaccine efficacy/effectiveness in low-income countries. An additional partially answered issue relates to rotavirus strain circulation in vaccinated populations. These issues are discussed in this review. The most imperative challenge ahead is to fulfill the WHO’s recommendation to introduce rotavirus vaccines in all countries.

## Rotavirus disease before the vaccine era

Rotavirus was first visualized in 1973 when electron microscopy was used to examine stools from ill children; subsequently, several decades of epidemiological research concluded that rotavirus was the main cause of acute diarrheal disease in children younger than 5 years of age worldwide ^1– 3^. Community- and hospital-based studies performed before 2006, the year current vaccines were licensed, proved that rotavirus was a key player in childhood gastroenteritis, accounting annually for 25 million outpatient medical visits, 2 million hospitalizations, and 400,000 deaths ^4^. At the time, best estimates indicated that every child would be infected by 5 years of age, one in 5 would require a medical visit, one in 65 would be hospitalized, and one in 293 would die, mostly children living in poor regions of the world.

## Rotavirus vaccines

### Vaccine development approach

Rotavirus vaccine development began soon after rotavirus discovery, and some key findings are summarized here:

i) Cohort- and daycare-based studies, aimed to define the natural history of rotavirus infection, demonstrated that repeated infections were common throughout the first few years of life but that it was the first infection that caused most moderate to severe symptomatic episodes, while subsequent infections tended to be milder or asymptomatic ^5, 6^. Thus, previous episodes “protected” against subsequent symptomatic episodes, less against reinfections in general, which could potentially be mimicked by vaccines.
ii) The discovery that humans could be infected by different rotavirus serotypes (strains that have non-cross-reacting neutralizing epitopes in the external VP7 and/or VP4 capsid when tested in cell culture) became a highly relevant issue during vaccine development. New serotypes are constantly emerging because of genetic reassortment, a process that is constantly occurring between animal and human strains. Nevertheless, over the last 40 years, fewer than 10 serotypes have been the predominant cause of childhood infections, indicating that only a few are fit to infect the human intestine. The predominance of a relatively small number of serotypes varies in an unpredictable manner between different regions and/or different time periods, most likely due to population-related immunity ^7^. From the 1970s to 1990s, a key question was what role serotype variability would play in vaccine protection. The fact that one antigenic serotype (for example, VP7 type G1), when inoculated into mice, did not elicit robust neutralizing antibodies inhibiting growth in cell culture of a virus with a different VP7 epitope (G2, G3, G4, or other) led to the concept of “homotypic immunity”, promoting a development strategy based on “multi antigenic component vaccines” ^8^. Child cohort studies, on the other hand, suggested that a multivalent vaccine approach could be avoided, as most children had at most one moderate to severe rotavirus infection, irrespective of the antigenic types to which they were exposed over the years ^5^. This observation promoted a vaccine strategy based on “heterotypic immunity”, where a single human attenuated strain could confer protection against different serotypes possibly by humoral and/or cellular-mediated processes other than VP7- or VP4-specific neutralizing epitopes ^9, 10^.
iii). Several humoral biomarkers have been correlated with protection against infection and disease in child cohorts evaluating natural infections and/or in early vaccine trials (serum and/or stool serotype-specific neutralizing antibodies, total IgA and IgG non-neutralizing RV-specific antibodies, and RV-secretory IgG and RV-specific T cells in animal models) ^11^. Consensus as to the specific protective levels of any of the above-mentioned biomarkers, suitable for use as a proxy for clinical protection, was not reached at the time and has not been reached to date. The lack of a biomarker obliged researchers to move forward with large efficacy trials based on clinical variables, of which moderate to severe rotavirus-positive diarrheal disease became the hallmark.

### Currently licensed vaccines

The multi antigenic component vaccine strategy based on animal-human reassortant strains was strongly promoted by scientists from the NIH, such as Al Kapikian, the “founding father” of rotavirus. This strategy led the vaccine development race during the 1990s, culminating in 1998 with the US licensing of the first vaccine, RotaShield®, a quadrivalent human-rhesus reassortant vaccine produced by Wyeth Lederle ^12^ ( Table 1). Vaccine efficacy studies had been promising, indicating protective rates ranging from 70 to 90% against moderate to severe gastroenteritis, measured by clinical scores including variables such as intensity and duration of diarrhea, severity of dehydration, fever, vomiting, and hospitalization ^13– 16^. Quite to the surprise of the rotavirus community, an early signal that the vaccine was associated with intestinal intussusception, an uncommon but severe event where the proximal jejunum telescopes into the distal portion causing acute intestinal obstruction, was confirmed after 12 months of vaccine use in the United States ^17^. Pre-licensure trials had hinted at the possibility; however, the studies were not sufficiently powered to demonstrate an increased risk, which was finally estimated to occur in roughly one out of every 4,670–9,474 vaccinated infants. The manufacturers withdrew RotaShield® and 8 years of further research were required before new vaccines reached licensing.

**Table 1.** Licensed vaccines and vaccine candidates in clinical phases of development.

Vaccine	Status	Comments	Selected references
RotaTeq®/Rotarix®	Worldwide license	Eleven years’ post-licensure; worldwide distribution; demonstrated effectiveness	Giaquinto *et al*. ^67^; O'Ryan *et al*. ^20^
Rotashield®	First licensed rotavirus vaccine in 1998 (USA); was withdrawn due to association with intestinal intussusception	Underwent a clinical trial with a two-dose regimen beginning within the first 30 days of life demonstrating 63% efficacy for the first 12 months of life	Armah *et al*. ^68^
LLR®/Rotavin-M1®/Rotavac®	Restricted license	Only used in China/Vietnam/India (respectively); lack of robust effectiveness data	Fu *et al*. ^32^; Dang *et al*. ^34^; Bhandari *et al*. ^69^
UK reassortant (Rotasiil®)	Restricted license	Phase III study	Isanaka *et al*. ^36^
RV3BB	Early clinical development	Phase I or early phase II studies	Danchin *et al*. ^70^; Luna *et al*. ^71^; Bines *et al*. ^38^; Naik *et al*. ^35^
Truncated VP8 subunit and a tetanus toxoid P2 protein	Early clinical development	Phase I/II study	Groome *et al*. ^39^

Adapted from 72

The vaccine race continued between two new candidates: 1) RotaTeq®, a multi antigenic component vaccine including five bovine-human reassortant strains, and 2) Rotarix®, a human attenuated single strain. Both candidates published their landmark phase III trial results in the same issue of *The New England Journal of Medicine* in January 2006 ^18, 19^. The efficacy of both vaccines against moderate to severe disease in middle- to middle–high-income countries, based on different clinical scores, surpassed 85%, and although many intended to advance efficacy comparisons, especially efficacy against different rotavirus serotypes, this was not possible, as a number of variables differed between the trials, including populations studied and primary and secondary endpoints. Unfortunately, comparative studies have not been performed to date. Importantly, pre-licensure efficacy and post-licensure effectiveness studies in developing countries have demonstrated that both vaccines are only about 50–60% efficacious against severe diseases, indicating that socioeconomic factors play a role ^20, 21^. Neither of the vaccines hinted at the possibility of an association with intussusception similar to RotaShield® in large clinical trials; these trials enrolled over 60,000 subjects, the sample size required to identify a 1:10,000 risk of intussusception. Nevertheless, post-licensure studies have demonstrated that both vaccines are associated with intussusception at a risk range of 1:20,000 to 1:100,000, a rate of risk that is considered a “class effect” ^22, 23^. The overall estimate of relative risk of intussusception in the 7 days following vaccination with Rotarix® and RotaTeq® was 5.4 and 5.5, respectively, following the first dose, and 1.8 and 1.7, respectively, following the second dose. The relative risk estimates were approximately tenfold lower than those reported for RotaShield® ^24^. This suggests that in a very small number of infants, possibly at increased risk for yet-undiscovered reasons for intussusception, the event may be triggered by vaccination, especially if the first dose is provided later into the first 6 months of life. This low risk needs to be acknowledged, although most recommending bodies clearly express that the benefits of rotavirus gastroenteritis prevention by far outweigh the low-level risk of intussusception, regardless of the geographic region in which the child lives ^25– 29^.

Four additional rotavirus vaccines, similar to RotaTeq® or Rotarix®, have been licensed: 1) Rotavac® and 2) Rotasiil® which are licensed by local manufacturers in India, with phase I–III supporting trials, 3) Lanzhou Lamb vaccine in China, and 4) Rotavin-MI® in Vietnam. The latter two vaccines were licensed with significantly fewer studies. Rotavac® includes the neonatal 116E rotavirus strain, a naturally occurring human-bovine reassortant strain of the G9P [11] serotype ^30^. In a phase III trial of nearly 7,000 Indian infants, protective efficacy against moderate to severe gastroenteritis of a three-dose regimen at 12 months of age was 56% ^31^. The Lanzhou Lamb vaccine, based on a rotavirus strain obtained in 1985 from a local lamb with diarrhea and attenuated through serial passages ^32^, was licensed in China in 2000. Despite the lack of studies on clinical efficacy and safety, over 30 million Chinese children under 5 years of age have been immunized using a schedule that includes a first dose for children 2 months to 3 years of age followed by annual boosters for up to four doses by 5 years of age. Effectiveness against rotavirus hospitalization seems to be around 60 to 78% ^32, 33^. Rotavin-MI® is similar to Rotarix® in that it is a G1P [8] attenuated strain obtained from a Vietnamese child. There is only one available published study on this vaccine that includes evaluations of different virus concentrations and doses in a phase I adult-infant and phase II infant trials ^34^. Rotasiil® is a UK bovine reassortant vaccine composed of five reassorted strains, with the added benefit of heat stability, developed in partnership with researchers from the USA, India, and Brazil ^35^. In Nigerien children, three doses had an efficacy of 67% against severe rotavirus gastroenteritis ^36^. Yet another vaccine, this time a quadrivalent vaccine, produced by Shantha Biologicals of India using the same bovine backbone strain, did not meet immunogenicity non-inferiority compared to the pentavalent vaccine, as anti-rotavirus IgA seroconversion was only 47% compared to 61% ^37^. A neonatal strain, RV3BB, developed by Australian researchers recently demonstrated a reasonable immune response in a rather small study; vaccine take occurred after three doses, regardless of whether the first dose was provided at 0–5 days or 8 weeks of life ^38^.

The only candidate based on an alternative strategy that has reached clinical trials is based on a truncated VP8 subunit protein of the human Wa strain and a tetanus toxoid P2 protein. In a phase I/II trial in children, immunogenicity against the homotypic antigen was high after three intramuscular doses but was significantly lower against heterotypic antigens, suggesting that this strategy will require a multicomponent approach ^39^. Interestingly, the vaccine had the effect of reducing subsequent live oral rotavirus vaccine shedding, suggesting some impact at the intestinal level ^40^.

## Rotavirus burden 10 years after rotavirus vaccine licensing

It is estimated that in 2015 there were nearly 2.4 billion episodes of acute diarrhea, of which nearly 950 million occurred in children younger than 5 years of age. In the same year, diarrheal diseases were responsible for nearly 1.31 million deaths, of which nearly 500,000 occurred in children under 5 years of age. Rotavirus was estimated to cause nearly 147,000 deaths. Between 2005 and 2015, the number of diarrhea cases in children under 5 years of age decreased by about 10%, and deaths due to diarrhea decreased by around 34%, while rotavirus deaths decreased by 44% (95% CI: 33–52%) ^41^. Attribution of this reduction in rotavirus cases and deaths to vaccine use is difficult, especially because vaccines are not widely used in the countries with the highest disease burden; nevertheless, rotavirus vaccines have most likely played an important role. As of January 2017, 92 countries have introduced rotavirus vaccines. This includes 85 national introductions, two ongoing phased introductions, and five subnational introductions ( http://rotacouncil.org/vaccine-introduction/global-introduction-status/). A dramatic decrease (>80%) in the incidence of severe rotavirus diarrhea has been reported in high-income countries, and a decrease of about 50% has been reported in low-income settings ^33^. Increasingly, evidence shows reductions in diarrhea-associated deaths of 31% in infants younger than 1 year old and 42% in children younger than 5 years old in countries with low child mortality ^42^. Specific data from low-income countries, where childhood mortality and specifically diarrhea-associated mortality is highest, are scarce. In South African children receiving two vaccine doses, Groome and colleagues showed 57% (95% CI: 40–68) effectiveness for rotavirus diarrhea requiring at least overnight hospital admission in children younger than 2 years of age ^43^. In Malawi children receiving two doses in an accelerated schedule, Naor Bar-Zeev and colleagues showed 64% (95% CI: 24–83) effectiveness for reduction of rotavirus-positive emergency room visits (compared with rotavirus test-negative controls) in children younger than 5 years old (94% of samples tested from children younger than 2 years of age) ^44, 45^. Furthermore, and most importantly, rotavirus vaccines have not been implemented in countries with the highest rotavirus-associated disease burden. Nearly 40% of sub-Saharan Africa and almost all South-East Asia (with the exception of India and Pakistan, where vaccines are being introduced in a phased format) have not yet introduced rotavirus vaccines.

## Challenges ahead

The most imperative current and future challenge is to fulfill the WHO’s recommendation to introduce rotavirus vaccines in all countries, with no exceptions. Funding and support priorities should and are being placed in countries with the highest mortality rates ^46^. Unfortunately, in many instances, a lack of funding is not the only limitation to implementation in these countries; there is also a lack of political will and/or recognition of the potential benefits of vaccination. In too many middle-income countries, where diarrhea-associated deaths are uncommon but where rotavirus-associated medical and emergency room visits and hospitalizations are significant, health authorities frequently fail to recognize the need for rotavirus vaccines. Increasing the importance of technical advisory groups, including the use of efficient decision-making tools (such as the Grading of Recommendations, Assessment, Development and Evaluations [GRADE] ^47, 48^), would help balance the viewpoints of health and financial authorities.

Live attenuated rotavirus vaccines have worked well, but we cannot consider current protection levels to be optimal. Unfortunately, efficacy and effectiveness decrease inversely with poverty. The reasons behind this phenomenon are not clear, but there is indirect evidence supporting several possibilities. Firstly, rotavirus infections occurred at younger ages and repeated symptomatic infections were more common in a birth cohort from a very poor area in India compared to a less-deprived area in Mexico ^5, 49^. This observation strongly suggests that viral exposure is significantly higher in poorer regions, most likely due to increased exposure to human feces. Vaccination at earlier ages, more doses, and/or higher dose concentrations may benefit such populations, but a significant increase in protection, from 50–60% to 80–90%, seems unlikely unless there is a concurrent improvement in environmental sanitation. Secondly, the co-administration of oral polio, more commonly used in developing countries, and rotavirus vaccines reduces the immune response to the latter, which most likely has some impact on reduced vaccine efficacy/effectiveness ^50^. A third factor may be increased incidence of breastfeeding in low-income areas. While breastfeeding did not significantly reduce the immunogenicity of rotavirus vaccine in Finish children, it was associated with a mild decrease in protection, especially during the second year of life ^51^. Mexican researchers demonstrated that breastfeeding can interfere with vaccine shedding and immune response ^52^. Thus, in poorer regions where breastfeeding tends to be more common, concomitant breastfeeding could play a partial role in reducing the efficacy/effectiveness of the vaccine by neutralizing vaccine replication to some extent. Three field studies addressing this issue provide good evidence that the role of breastmilk in reducing vaccine immune responses is non-existent or minimal. In a well-designed trial in Pakistan, rotavirus IgA seroconversion rates and geometric mean titers were evaluated in vaccinated children randomly assigned to one of two groups, a group where breastfeeding was withheld for at least 1 hour before and after vaccination and a group where infants received at least 20 minutes of breastmilk at most 10 minutes before vaccination. Rather surprisingly, IgA seroconversion rates were roughly 10% higher in the group receiving breastmilk after both the first and the second dose. Hints of possible interference were observed in the antibody titers achieved, which were lower among seroresponders receiving breastmilk after the first dose compared to those not receiving milk; a subset of infants with low maternally derived antibodies receiving high-antibody-containing breastmilk seemed to have a reduced immune response ^53^. A similar study in Indian children in whom breastmilk was withheld for 30 minutes before and after vaccination, or encouraged, showed no differences in seroconversion rates; in this study, seroconversion rates where quite low in both groups (26–27%) ^54^. In a South African study with seroconversion rates near 60%, withholding breastmilk for 1 hour showed no difference compared to a breastmilk-encouraged group in anti-rotavirus IgA seroresponse rates or antibody titers achieved ^55^. Other factors are possible, such as the increased prevalence of severe malnutrition leading to reduced vaccine immune responses; however, this hypothesis is supported by only one relatively underpowered study ^56^.

Environmental enteropathy is a subclinical condition characterized by small intestine inflammation with shortened villi, intestinal barrier dysfunction, and reduced nutrient absorption. This condition seems to be common in children living in poor, unsanitary conditions and is thought to be caused by repeated or chronic exposure to enteropathogens and by malnutrition ^57^. Studies using biomarkers associated with this condition show that it is present in over 80% of 12-week-old infants in Bangladesh ^58^. Using a complex model, this condition was reported to be associated with decreased seroresponse and failure of the vaccine Rotarix®. In children from El Salvador, this condition was also associated with lower seroresponse rates to RotaTeq® ^59^.

Lastly, differences in the gut microbiota/microbiome have been proposed as a factor affecting vaccine effectiveness; while regional differences exist ^60^, the potential that the gut microbiome plays any role in differential protection rates will require future studies, which are currently underway ^61^. A recent publication is enlightening, as it shows that children from Ghana responding to rotavirus vaccination as determined by an anti-rotavirus IgA titer >20 IU/mL have a different microbiome profile compared to non-responders. Interestingly, responders had a microbiome profile more similar to a Dutch infant group compared to non-responders ^62^.

Serotype replacement leading to an increase in non-vaccine serotypes over time, due to possible vaccine selective pressure, has been repeatedly postulated during the past decade, but it lacks robust supporting evidence. Increases in the predominance of specific rotavirus serotypes, heterotypic to the vaccines in use, have occurred, but mostly in a temporal manner similar to the known unpredictable regional and temporal variability of rotavirus serotype circulation observed before the vaccine era ^7, 63^. The biological plausibility of a selective pressure phenomenon is low, as rotavirus vaccines do not abolish the circulation of any particular serotype (protection against infection does not surpass 60%) and “competition” between different serotypes within the intestine (as occurs for pneumococcus in the nasopharynx) is unlikely. Low-level emergence of “uncommon serotypes” such as G5, G8, G12 ^64^, and the more significant G9 serotype, which emerged as a new, frequently predominant serotype during the past few decades, are ongoing phenomena which will most likely continue to occur with or without widespread vaccination. Because different vaccines are most likely not equally protective against all potential human serotypes, and because some novel serotypes may be more fit for the human intestine, it is possible that one or more of these less common types could prevail over others (relative predominance) for a given time period. Importantly, 10 years after vaccine introduction, the emergence of uncommon strains has been mild. A well-performed systematic literature review and meta-analysis including publications with content on rotavirus vaccine effectiveness and strain characterization published from January 2006–2014 concluded that vaccines have similar effectiveness against partly or fully heterotypic strains compared with homotypic strains. It also concluded that the emergence of particular serotypes has not occurred after vaccination ^65^. Serotype surveillance will continue to be important, especially in low-income countries, where vaccines are less efficacious and data on rotavirus serotype distribution are scarce; importantly, the interpretation of serotype variations should be carried out with caution.

Further considerations of parenteral protein-based rotavirus vaccine candidates, if proven safe and efficacious, may be of benefit for several reasons. First, we could move away from any risk of intussusception, as it is unlikely that a parenteral vaccine would be a trigger. Second, theoretically any external interference with vaccine take (maternal antibodies, breastmilk, and live poliovirus) would be unlikely, with the potential for increased efficacy, although this is highly speculative at the moment. Third, a combination vaccine with another major cause of diarrhea, such as norovirus, could be considered; one current norovirus VLP and rotavirus VP6 nanostructure-based vaccine demonstrated an interesting adjuvant effect of the rotavirus component on norovirus immune response ^66^, although it is unclear if this would provide protection against rotavirus. Combination with rotavirus outer capsid proteins ^39^ may be a future avenue to explore.

Forty-five years after rotavirus discovery, extensive research efforts have led to safe and effective vaccines, which are reducing childhood deaths and suffering. It has been a success story, which is not over. Several pending issues have been discussed here, and the next decade should bring new insights, advances, and answers and, most importantly, significantly more children receiving rotavirus vaccines.
